# The Relation of Social Life to Surgical Disease

**Published:** 1888-11

**Authors:** D. Hayes Agnew


ARTICLE II.

### THE RELATION OF SOCIAL LIFE TO SURGICAL DISEASE.

---

PRESIDENT'S ADDRESS.

DELIVERED BY D. HAYES AGNEW, M. D.

---

[President of the American Surgical Association, September 18th, 1888.]

*Fellows of the American Surgical Association:*—As the generations fare on, enriched by the results of scientific labor, which pours its tides of opulence into all departments of human thought and industry, there follow, through a reactive or reflex influence, certain notable changes, not only on the life and manners of a people but on their physical and mental diseases. The more advanced a civilization the more complex become the problems which surround it. While the accumulation of wealth and the multiplication of appliances for human comfort have, in the aggregate, contributed to the well being of the race, yet there is reason to fear that the insatiate and ambitious demands of the masterful leaders in the work of the world, unless conditioned and environed by reasonable safeguards, may acquire their triumphs at the expense of human life. It is a suggestive and a solemn thought, that in the victorious march of civilization, thousands of victims must perish beneath her chariot wheels. There really seems to be a perpetual antagonism between man's inventions and discoveries and the well being of a no inconsiderable fraction of humanity. He reduces the elastic vapor of water to practical use, and is rewarded by seeing countless numbers of human beings blown into shapeless masses by his rebellious servant. His chemistry creates formidable explosives capable of dislodging the solid strata of the earth, and these, in wicked hands, become instruments for consummating such diabolical plots as serve to unsettle the peace of a nation. He rears manufactories for fashioning multitudinous fabrics which minister to the comfort and luxury of the race; and yet while the hands of the fabricator are busy manipulating the materials of these industries, he is breathing a death-laden air. We send our missionaries to China and the Sandwich Islands to reclaim their peoples from the barbarities of heathenism, and then our commerce to ruin their souls and wreck their bodies. There seems, indeed, to be an eternal conflict between good and evil.

Considerations like these naturally lead to a very inviting field of study, namely, the relation between the material prosperity of a people and the forms of their disease. What I propose, however, in discharging one of the duties belonging to the honorable office to which, by your kind suffrages I have been elected, is very briefly to follow one line of this inquiry, that is—

THE RELATION OF SOCIAL LIFE TO SURGICAL DISEASE.

There is no tyranny more exacting or despotic than that exercised by the conventionalities which govern our living. All stages of life from infancy to old age are under its domination. It dictates the education, the manners, the walk, the dress, the forms of speech, in fine, the whole being. Beyond all contradiction the behests of fashion are vastly more influential in governing public conduct, than any arguments drawn from the teachings of structure and function. As a rule, when the conflict is between taste and reason, the victory will be on the side of taste. In nothing is this more favorably displayed than in the apparel used to protect the body.

It is not an agreeable task to peer into the wardrobes or dressing rooms of our fair countrywomen. I have no special taste for exploring museums of bizarre collections; indeed, without a key to interpret the curious and ingenious mechanisms for clothing the form divine, such an exploration would be like an archaeologist attempting Egyptology ignorant of cuneiform inscriptions. I have, however, some knowledge of human anatomy in its broadest sense, and when I look upon the masterpieces of the human form, whether in marble or on canvas, a Belvidere Apollo or a Venus de Medici, and contrast these with the dressed out specimens of modern women, I am forced to admiration; not so much at the amazing ingenuity displayed in concealing the divinely appointed form, as at the plasticity and patient submission of mortal clay under the despotism of a conventional inquisition. Were these processes of mutilation and abnormality harmless, did the body consist of a mere mass of protoplasm, capable, under the application o certain stimuli, of assuming, normally, protean shapes, the subject might be passed over with the feelings of a naturalist; but this is not so. These violations of the laws of structure bring with them serious penal inflictions, which, did they terminate with the original offender, might be dismissed with a sentiment of pity; but projecting, as they do, their baneful consequences to successors, they become proper subjects for criticism.

Let me name a few examples as illustrative of my subject. For some time the profession has been speculating on the causation of nasal and post-nasal catarrh, with its accompanying auditory defects, the growing frequency of which cannot have escaped general observation. Doubtless no single agency will explain the presence among us of this unpleasant disease, yet there are facts connected with this affection which to me are very suggestive. I cannot recall an instance in which I have met with the disease among females belonging to the Society of Friends, Dunkards or Mennonites. If this, on more extended observation, proves to be true, may not the head-dress peculiar to these people be accepted in explanation of their exemption. The bonnet, which at one time overshadowed the entire head, as all know, has been gradually shrinking in its dimensions, until it has become a mere shadow of its former self, and offers no protection whatever to the head. As a substitute, I would not insist upon the quaint head gear of the Friend, though I believe that any modification which will protect this part of the body will lessen the tendency to catarrhal inflammation of the naso-pharyngeal mucous membrane.

### MUSCULAR RESTRAINT.

A legion of physical imperfections arise from muscular restraint. Among these may be mentioned weak ankles, narrow or contracted chests, round shoulders, projecting scapulæ and lateral curvatures of the spine. The foolish concession to appearance and unwise partiality of parents for enforced systems of education, the demands of which bear no just proportion to the capacity of the infantile mind, constitute the initial or determining force of these physical imperfections. In many cases the weak ankles of children, characterized by eversion of the feet, thus allowing the superincumbent weight of the body to be transmitted to the latter inside of the proper centre of support, is largely chargeable to the miserable practice of placing on the little ones, long before they are able to walk, boots tightly laced up the limb some distance above the ankles. The confinement of the flexor and extensor muscles by this conscriction prevents that free play of movement which reacts so favorably on all the elements of an articulation, and that, too, at a time when the growing forces are at full tide, so that when the time arrives for standing and walking, the muscles are unequal to the firm support of the joint. The consequence of this feebleness is soon seen in the turning outward of the feet, throwing the strain on the internal lateral ligaments, which in turn become elongated through growth, and thus the defect becomes established; but the evil does not terminate here. The calcaneo-cuboid and the astragalo-scaphoid ligaments, losing the proper support of the tendon of the posterior tibial muscle, under the abnormal tension begin to yield, and to the deformity of eversion is added that of "flat-foot." That the above is not a mere hypothetical explanation of the ankle defects, I have many times verified by finding the threatening symptoms disappear after liberating the imprisoned muscles and subjecting the enfeebled parts to a judicious massage. Under no circumstances, as is too often the case, should instrumental apparatus be applied, unless in cases where, from neglect, the deformity is thoroughly established and is progressive.

Take another deformity, that of bow-leg. On the earliest signs of the unsightly curve, the limb is too often trammeled with irons, and the growth of the muscles arrested, when it is well known that if manual force be systematically applied two or three times a day, the limbs will gradually assume their typical form.

Again, in further illustration of our general text take as an example a child who, for one long or two short sessions, for six days of the week, sits over the study desk, compelled to assume a position in which, from the inclination of the body, the shoulders fall forward, the head being supported most probably on the elbows and hands. In such a posture the great serrati and pectoralis major and minor muscles are in a state of relaxation, while the erector spinæ and trapezei muscles are in a state of tension. This change in the position of the shoulders gives the scapulæ over, without antagonism or resistance, to the action of the rhomboidei and the levatores angulæ scapulæ muscles, which, acting conjointly, cause that projection of the lower angles of the shoulder blades which the older anatomists termed "scapulæ alatæ." To all this must be added the very important factor of four to six hours in the school room and two hours, at least, of home preparation for the following day's recitations, during which time the respiratory functions having been reduced to a minimum of activity, the muscles of the chest are comparatively passive and areation of the blood tardy. Certainly no combination of conditions could be better devised for forming contracted chests and round shoulders. It is not long before the watchful eye of the mother detects the change in the figure of her child. She will probably discover this and take alarm, even when the pale face, the languid air and the capricious appetite of the child cause no anxiety; and then comes the second act in the drama of physical deterioration, namely, a resort to shoulder braces and stays, in order to accomplish that which the muscles should be taught to do without restraint or incumbrance.

### LATERAL CURVATURES.

While it is true that lateral curvatures of the spine depend upon causes both central and peripheral, yet in no small number the deformity is clearly attributable to influences of a social nature. The young column, by reason of the non-union of the epiphysis and diaphyses and the supple character of its ligaments, is extremely flexible. Whatever, therefore, destroys the muscular equipose, however inconsiderable the force, if persistently repeated, changes the centre of gravity and develops primary and compensating curves. For six months, or over, in the year, any fine morning, groups of children may be seen plodding along our streets with a miniature library of books suspended from one shoulder. To the already preponderating scale of the balance, add this additional factor, a probably badly arranged light, compelling these little *savants* to assume a lateral inclination of the body in order to obtain the necessary illumination of the subject of the study, and you have all of the conditions necessary for perpetuating the lateral deformity. "Just as the twig is bent the tree's inclined." As in the case of round shoulders, so here, in order to prop up the falling column, instrumental contrivances are immediately called into requisition. The body is encased in a formidable coat of mail, to be followed by muscular atrophy and permanent distortion of one of the otherwise most beautiful pieces of mechanism in the human frame. It is true that in most educational institutions for the young provisions are made for physical culture, and these are in some measure antidotal to the evils complained of, but, in my judgement, do not at all compensate for that free, unstudied romp in the open air, untrammeled by the hard and fast rules of calisthenics, so fascinating to the young child. Nor does the evil end here. While the forcing process which is to stimulate the mental powers far beyond the real capacity of the immature and growing brain to receive is in progress, another is inaugurated, which is to qualify especially the female child to acquit herself with distinction when the time arrives for entering the great world of society, or as Thomas Brown would style it, "for the frivolous work of polished idleness." The gait and carriage must be reduced to prescribed rules, the voice toned down to a drawl or trained to move like a mountain torrent. The muscular apparatus of the face must be taught to express, not the spontaneous and natural outflow of feeling which wells up unbidden from the magic chamber of the heart, but rather to produce an effect; and so this work of transformation goes on until it culminates in the full-blown society girl. Is it any wonder that, under such a scheme of education, conducted throughout by a studied disregard of both the physical and mental constitution, and exercising as it does such tremendous drafts on the nervous system, the world is becoming filled with a class of flat-breasted, spindle-limbed young women, unfitted for the varied and responsible functions of womanhood; qualifications, too, which under a different regimen and directed into proper channels would exert a most potential influence on all the great social and moral problems of the age.

While thus plain spoken on the frivolous methods of living, I do not wish to be understood as being unfriendly to the highest cultivation of the mental and physical powers, if conducted on lines in harmony with the organization, nor to any technique which may conduce to personal grace or elegance of manners, so that the manly or womanly personality of the individual be not sacrificed to the Moloch of sentiment and sham. Indeed, indifference to these things is inexcusable, in either man or woman, as not only lessening their influence in the world, but, in many respects disqualifying them for the highest discharge of the duties of modern life. Valuable as may be the unpolished diamond, yet it is only after the wheel of the lapidary has worn away the dull incrustations that its true brilliancy is revealed and the gem is fitted to adorn the breast of beauty.

### BODILY CONSTRICTION.

In the further discussion of my subject, I may next notice the evils of visceral displacement and pressure, consequent on abdominal constriction. Whatever may be said in regard to Greek and Roman life, the infinite care which these people displayed in developing and maintaining the very best type of the human form, is worthy of admiration. The Ionic "cheton," spoken of by Attic writers, and so often represented in the bronzes of Herculaneum, while it would not exactly satisfy the modern idea of dress, was at least free from the charge of interfering with the contour of the human figure. The painters and sculptors of those classic days were reverent students of nature. Their delineations were true to life. Their works furnish us with no hour-glass contractions of the human body. The constriction of the waist operates injuriously on both the supra- and infra diaphragmatic organs. Any force acting on the base of the thorax and preventing the expansion of its walls concentrates the function of respiration, which should be general, on the apices of the lungs, and hence, under these circumstances, the movements of breathing are for the most part confined to the summit of the chest. As the initial seat of tuberculosis is located at the upper part of the lungs, may not the inordinate work entailed on these parts by constriction have some part in hastening such deposits in the female, where the predisposition exists? It is this forcing inward of the costal border of the thorax which causes the groove on the anterior surface of the liver, so familiar to anatomists, This pressure cannot fail to interfere with the descent of the diaphragm, and with the functions of the gall bladder and duodenum, and exercises no small degree of influence in favoring the formation of biliary calculi, females being peculiarly prone to such concretions. The extent to which the liver may be damaged by extreme constriction of the waist, is well illustrated by a case quite recently reported in the *British Medical Journal*, in which a considerable portion of the left lobe of the liver had been separated from the right, the two being connected only by a band of connective tissue, and which enabled the operator to remove the detached mass without difficulty. The evil effects of this constriction on the viscera of the abdomen and pelvis is most strikingly witnessed in the embarrassed portal circulation, in the different uterine displacements, elongation of ligaments, displaced ovaries, tubal inflammations, hemorrhoids, herniæ and other morbid conditions which either prevent or disqualify the woman for the exercise of those functions of maternity, and which in addition, through reflex influences, entail a host of functional disorders reaching into every avenue of the body and invading both the mental and moral constitution of the victim. So prolific have these infirmities become that a new department of surgery has been organized for their special management. To what, if not to social causes, can these morbid changes of structure in the pelvic organs, especially of the uterus and its appendages, be attributed? Why should laceration of the cervix uteri be so common an accident? Labor is a natural process and ought not, under ordinary circumstances, be attended by lesion of uterine tissue. I can conceive of no agency more likely to induce that muscular degeneration which predisposes to this accident than the modes and methods of modern living, especially among the inhabitants of great cities. In the expression "modern living" much is embraced. It includes culinary pharmacy, over-feeding and drinking, insufficient or injudicious exercise, improperly heated apartments and a disproportion between the hours of exercise and rest. Contrast if you will the muscles of the hardy country housewife, who, bearing the cares and responsibilities of a dependent family, bustles about the livelong day in-doors and out-of-doors, eats with a relish her plain and simple fare, repairs at reasonable hours to bed and sleeps the sleep of the beloved, undisturbed by dyspeptic nightmares, and rising with the golden dawn, resumes the round of domestic toil with a clear head and supple limbs. I say contrast this type of a class with that of another, the woman born to luxury and ease, whose capricious and exacting taste taxes the art of the professional caterer, who drags out the morning hours toying with some crazy pieces of embroidery or trashy novel, lunches at one, rides out in the afternoon for an airing of two or three hours, returns to a dinner of five or six courses at seven, completes the evening at the opera, the theatre or the assembly, and coming home after midnight, crawls into bed weary and exhausted in body and mind, only to rise with the best hours of the morning gone, for another day of aimless routine life. Can it be doubted that in the first case, with a digestion unimpaired, with the products of textural change consumed by functional activity and eliminated through the proper emunctories, the woman should possess a vital resistance and a tone of tissue altogether superior to that of the other, whose habits of living must necessarily favor their faulty metamorphosis ?

To these same agencies must be attributed that brood of nervous and hysterical evils for the relief of which the gynæcologist too often, I fear, invades the domain of womanhood, around which her whole sexual nature revolves, and which, save only in the direst extremity, should be sacred against all operative intrusion.

LATE MARRIAGES.

constitute another social evil, the penal inflictions of which involve both sexes alike. Pride and luxury determine long engagements or deferred proposals. Marriage, it is believed, necessarily involves an establishment, a display, a retinue of servitors. The good old notion of two souls being united in wedlock for the purpose of being mutual helpmates, and patiently together working up from modest beginnings to affluence, seems to be entirely at variance with the modern idea of this relation. In the meantime the young man is betrayed into unlawful sources of gratification, alike destructive to moral and physical purity, the pollution of which incontinence is often subsequently communicated and perpetuated to wife and offspring. I would not dare to say how many cases of this nature have been entrusted to my professional confidence, though I doubt not my experience does not differ from that of many of my professional brethren whom I address. It is under such circumstances that many of those infective inflammations of the Fallopian tubes, as salpingitis and pyosalpinx, arise, and which entail the most serious deterioration of health.

THE FOOT AND THE SHOE.

It may be thought by some persons that the subject of the foot and the shoe is not of sufficient dignity to appear in a public address. The Romans and the Greeks thought differently. The literature of both people is full of references to the shoe worn by both sexes. So important, indeed, are the feet to the well-being of the body, that whatever impairs their usefulness, either for support or locomotion, becomes a positive calamity. Nothing can be more unlike the human foot than the modern shoe. Let any one leave the impress of his or her foot in the wet sand of the sea-shore, and then place alongside of the imprint a fashionable shoe; that the two were ever intended for each other would scarcely strike a child of the forests. The North American Indian entertains juster notions about clothing this portion of his body than does the civilized citizen of New York and Philadelphia. Compare the moccasin with the shoe of the city belle. Compare the foot-gear of Pollux and of Aristophanes with the same, and we shall see that the savage and the polished Greek alike understood the value of sound feet in the race of life. It is the imperfect adaptation of the shoe to the foot which constitutes the fruitful source of the tired ankles, corns, bunions, over-lapping of the toes and ingrowing nails. Some idea may be formed of the magnitude of the evil from the fact that of eight hundred patients under the care of a prominent chiropodist of Philadelphia, the great majority of the defects were entirely attributable to the high heels, and the contracted toes of the shoes. Especially do these physical encumbrances arising from a blind submission to social laws operate disadvantageously to our fair women at the beginning of the new dispensation, requiring both muscles and brains, and when her friends propose to sweep away all the old traditions, and claim for her the earth with all its masculine employments.

GAMES AND AMUSEMENTS, which in themselves are proper and praiseworthy, too often become developed into a craze, working both moral and physical mischief. Professor Leuf, himself a professional in the national game of baseball, has described the pitcher's arm, a condition of over-taxed function, and one in which all the anatomical elements of the upper arm are involved. There is also the tennis arm and the swollen, super-sensitive prostate of the bicyclist, both due to the abuse of popular amusements.

Defects of refraction or visual defects constitute another class of affections fairly attributable, in many instances, to social influences. The number of children which may be seen on our streets any day wearing glasses, has become a matter of common observation. It is far from being probable, that the most exquisite piece of mechanism, the human eye, came from the Divine Artificer imperfect. Because eyes are young, it does not follow that they are thereby better fitted to sustain prolonged use. Just the reverse is true, and it is high time that parents and educators begin to recognize the fact. The power of the eyes for continued use, like that of other organs of the body, is one of gradation. It moves in the general procession and strengthens with the advance in life, until development has attained to its zenith. Not only so, but the eye being a part of the body, it must suffer or rejoice through the operation of general causes. A bone may have its normal curves changed, a tendon may slip from its appointed groove, or a blood vessel be destroyed, and yet very little disability be realized ; but the eye is made up of such extremely delicate structures, and so acts according to fixed physical laws, that not the slightest alteration of a curve, or the mobility or density of its media, can occur without great vitiation of function. To exact, therefore, long hours of study from children of a tender age, involves a degree of functional strain altogether disproportionate to the structural resources of the organ; and by disturbing the orderly processes of nutrition gives rise to hypermetropia, asthenopia, astigmatism, and its companion headache. That the picture is not too highly colored or the causation overstrained, we have only to contrast the children born and reared in those portions of the country not too much dominated by the methods of modern civilization, and who rarely demand a resort to artificial aids to provide for abnormalities of vision. The only remedy for the evil when infantile scholarship is insisted upon is the Kindergarten or object lesson system, the most natural and effective plan of impressing the young mind.

RENAL DISEASE.

Is there any reasonable explanation drawn from sources of a social nature for the great frequency of those renal disorders which come more particularly under the care of the surgeon, as crystalline deposits and calculi? For maintaining the general health at the highest phisyological standard, a proper quality of food and the proper disposal of tissue waste are essential conditions. Along with wealth and luxury come the abuses of the table. Americans are fast becoming a nation of dyspeptics. Our country is so rich in the products of every zone, that nowhere else in the world can you find such a variety of food, animal and vegetable. These foods manipulated in a thousand ways by the subtle art of the professional cook, almost necessarily betrays one into excess, and also creates the desire for wines and other alcoholic beverages to aid the stomach in disposing of its plethoric supply. In great cities, which furnish relatively the largest number of cases of renal disease, affecting pre-eminently the mercantile and sedentary classes, we find just the conditions favorable to their development. The competitions of trade keep the merchant always at white heat. Time is golden, and the street car and other means of conveyance annihilate distance, and the ride is substituted for the needful walk. A hasty lunch at the most convenient restaurant satisfies the inner man, until the business of the day is closed, when, weary and worn, he is driven to his home to partake of a course dinner, the balance of the evening to be spent on the lounge with the evening paper or the latest periodical. To the literary man the fascinations of the study and the library charm him away, with their siren voices, from the fields and the highways, until bodily exercise grows distasteful and repugnant. In the meantime there has been no provision made for the waste or tissue metamorphoses of the body through that great agency exercise. These accumulate in the blood, the internal eliminating organs, of which the kidneys are chief, are overtaxed, and then follow the evils of mal-assimilation, and of excretion, in the form of urates and oxalates, often resulting in the formation of calculi.

In conclusion, may we ever hope for a time when the race will realize that these bodies which we wear, which God has so highly honored by his own incarnation, are sacred temples, to be kept in harmony with recognized physical laws, and not to be made instruments of mere animal gratification ?—*The Polyclinic.*

